# TXNIP: A key protein in the cellular stress response pathway and a potential therapeutic target

**DOI:** 10.1038/s12276-023-01019-8

**Published:** 2023-07-03

**Authors:** Eui-Hwan Choi, Sun-Ji Park

**Affiliations:** 1 New Drug Development Center, Daegu-Gyeongbuk Medical Innovation Foundation (DGMIF), Daegu, 41061 South Korea; 2 Division of Nephrology, Department of Medicine, Washington University School of Medicine, St. Louis, MO 63110 USA

**Keywords:** Inflammasome, Experimental models of disease, Stress signalling, Mechanisms of disease

## Abstract

Thioredoxin-interacting protein (TXNIP), which is also known as thioredoxin-binding protein 2 (TBP2), directly interacts with the major antioxidant protein thioredoxin (TRX) and inhibits its antioxidant function and expression. However, recent studies have demonstrated that TXNIP is a multifunctional protein with functions beyond increasing intracellular oxidative stress. TXNIP activates endoplasmic reticulum (ER) stress-mediated nucleotide-binding oligomerization domain (NOD)-like receptor protein-3 (NLRP3) inflammasome complex formation, triggers mitochondrial stress-induced apoptosis, and stimulates inflammatory cell death (pyroptosis). These newly discovered functions of TXNIP highlight its role in disease development, especially in response to several cellular stress factors. In this review, we provide an overview of the multiple functions of TXNIP in pathological conditions and summarize its involvement in various diseases, such as diabetes, chronic kidney disease, and neurodegenerative diseases. We also discuss the potential of TXNIP as a therapeutic target and TXNIP inhibitors as novel therapeutic drugs for treating these diseases.

## Metabolism: A protein target for diverse disorders

A protein with the potential to fuel uncontrolled inflammation and cell death could offer a target for a variety of metabolic, neurodegenerative, and other diseases. The cellular redox system is a set of reactions that helps prevent accumulation of toxic byproducts of metabolism. Stressful conditions lead to the activation of the TXNIP protein, an inhibitor of the redox system, and Eui-Hwan Choi and Sun-Ji Park at the Daegu-Gyeongbuk Medical Innovation Foundation, South Korea, have reviewed how TXNIP contributes to diverse pathophysiological states. For example, TXNIP-mediated redox inhibition is associated with the nervous system inflammation seen in Alzheimer’s disease, and also contributes to premature death of insulin-secreting cells in patients with diabetes. Several drugs have been identified that can reduce TXNIP activity, and ongoing preclinical studies are now examining the therapeutic potential of such agents.

## Introduction

Thioredoxin interacting protein (TXNIP), which is also known as thioredoxin (TRX) binding protein-2 (TBP-2) or vitamin-D3 upregulated protein 1 (VDUP-1), was identified as a binding partner of TRX in a yeast two-hybrid screen^1,2^. TXNIP was originally discovered in HL-60 cells as a 1,25-dihydroxy vitamin-D3 responsive gene, hence its original name of VDUP-1^1^. TXNIP has an α-arrestin domain that interacts with and reduces the activity of cytosolic TRX and mitochondrial TRX (activated form), thus controlling cellular redox signaling^3^. TRX helps sustain the activity of the TRX system through a redox mechanism. The TRX system regulates the preservation of a reduced cellular environment and includes TRX reductase, nicotinamide adenine dinucleotide phosphate (NADPH), and TXNIP^3,4^. TXNIP activates TRX as a negative regulator to maintain the redox balance and is a core mediator of proliferation and apoptosis. Moreover, this factor is also linked to the development of TXNIP-related metabolic diseases such as diabetes^3–5^.

TXNIP plays an essential role in metabolism maintenance by activating inflammatory signaling via the nucleotide-binding oligomerization domain (NOD)-like receptor protein-3 (NLRP3) inflammasome. The inflammasome is composed of three components: NLRP3, the caspase recruitment domain (ASC), and pro-caspase 1^6^. Inflammasome activation is initiated by the interaction between TXNIP and NLRP3. The generation of reactive oxygen species (ROS) induces the interaction of NLRP3, ASC, and pro-caspase 1, resulting in the formation of inflammasomes and the secretion of interleukin 1β (IL-1β) and IL-18, thus facilitating the inflammatory reaction^7^. TXNIP is considered a critical link between endoplasmic reticulum (ER) stress and inflammation. TXNIP induction by ER stress activates NLRP3 and triggers the formation of the NLRP3 inflammasome complex^7,8^. Furthermore, ER stress and oxidative stress can induce subcellular shuttling of TXNIP to mitochondria, which triggers ASK1-induced mitochondrial apoptosis^9,10^.

Several studies have identified TXNIP upregulation in diseases such as kidney disease, neurodegeneration, and type 1 and 2 diabetes. TXNIP modulates the transcription of several genes, indicating the involvement of various mechanisms that implicate a new function of TXNIP. Thus, considering the upregulation of TXNIP in numerous diseases, TXNIP could be a potential drug target for therapeutic development. In this review, we summarize the known roles of TXNIP in the regulatory mechanisms underlying oxidative stress, ER stress-mediated NLRP3 inflammasome activation, pyroptosis, mitochondrial stress-induced apoptosis, and the pathogenesis of diabetes, chronic kidney disease, and neurodegenerative diseases.

## Multiple functions of TXNIP in the cellular stress response pathway

### The TRX system: TXNIP regulates oxidative stress

TXNIP is a central physiological inhibitor of the TRX redox system, and its transcription and activity levels play a major role in redox regulation. The redox system is composed of TRX, TXNIP, NADPH, and thioredoxin reductase (TRX-R)^11^. TRX is present as different isoforms, including cytosolic TRX1 (12 kDa) and mitochondrial TRX2 (15.5 kDa). TRX1 is the primary TRX isoform, is widely distributed in the cytosol, nucleus, and plasma membrane and is even secreted into the extracellular space under certain conditions^4,11^, while TRX2 is specifically localized. TRX reduces oxidized proteins, resulting in the oxidation of 2-cysteine residues and constant alternation between its oxidized (inactivated form) and reduced states (activated form)^4^. Oxidized TRX is converted to its reduced state by NADPH-dependent TRX-R activity via the catalysis of electron transport from NADPH to oxidized TRX, thus controlling intracellular redox balance^12^. Compared to wild-type mice, mice with TRX1 overexpression exhibit increased resistance to oxidative stress, cardiomyocyte injury suppression, and lung cancer ablation via the inhibition of ROS production, suggesting protective effects of TRX^13^.

The essential physiological role of the TRX system is to protect cells from oxidative damage and maintain a reduced intracellular microenvironment by removing intracellular ROS via disulfide reductase activities^14^. ROS are usually generated by cellular metabolism involving mitochondria, and it is important to regulate optimum levels of ROS in cellular processes involving signal transduction and gene transcription. However, excess accumulation of ROS during cell proliferation can lead to lethal damage to cellular components, such as DNA, RNA, and proteins, which in turn activate cell death programs^15^. Thus, efficient intracellular antioxidant systems that balance ROS generation and scavenging to sustain redox homeostasis are indispensable for cell metabolism.

TRX1 and TRX2 regulate cytoplasmic and mitochondrial ROS levels, respectively. However, TXNIP suppresses the antioxidant activity of TRX. Mesangial cells from TXNIP-knockout mice (Hcb-19) under high glucose conditions generated two- to threefold less ROS than wild-type cells (C3H), suggesting the inhibitory effect of TXNIP on the redox function of TRX^16^. In addition, Yodoi et al. showed that transient overexpression of TXNIP in HEK293 and COS-7 cells inhibited the reducing activity of TRX1 and downregulated its expression at the transcript and protein levels^17^. Thus, TXNIP is a pro-oxidant that increases ROS generation and induces oxidative stress. TXNIP binds specifically to reduced TRX (TRX-(SH)2, activated form) and not to oxidized TRX (TRX-S2, inactivated form) at the catalytic site^18^. The interaction of TXNIP and reduced TRX via active catalytic sites requires dithiol-disulfide exchange reactions at the redox-related cysteine residues on each protein and induces structural reorganization of TXNIP^18^. TXNIP has an intramolecular disulfide bond between cysteine residues (Cys) 63 and 247, which is essential for its interaction with Cys 32 of TRX. Studies have identified redox-dependent and redox-independent effects of TXNIP and the production of a C247S (TRX binding site) mutation in TXNIP that cannot bind TRX^19^. TRX regulates TXNIP function as a lipid inhibitor by improving its stability rather than TXNIP regulating TRX. This distinct complex of TRX and TXNIP, which is also known as the redoxisome, primarily regulates the ROS signaling pathway (cytosolic and mitochondrial)^3,4^.

In the physiological state, TXNIP is localized to the nucleus and cannot translocate to the cytoplasm^10^. Under excessive ROS conditions, TXNIP expression is upregulated by inhibition of the phosphorylation of adenosine 5′-monophosphate-activated protein kinase (AMPK) and the translocation of TXNIP from the nucleus to the cytoplasm, thereby resulting in ER and mitochondrial stress^20^. Nuclear to cytoplasmic translocation of TXNIP promotes the interaction between TXNIP and TRX1, leading to the inhibition of TRX1 activity and resulting in the development of various diseases. Antoine et al. showed that decreased TRX expression due to aortic TXNIP overexpression was associated with endothelial dysfunction related to arterial aging and increased expression of NADPH oxidase^21,22^. A reduction in aortic TXNIP expression levels increased TRX expression levels, leading to the ablation of NADPH oxidase expression^21^, which protected against endothelial dysfunction induced by several disorders. Research on diabetes-related mouse models has demonstrated the generation of oxidative stress and excessive ROS production due to the ablation of TRX activity by TXNIP^23^. In addition, TXNIP regulates the production of ROS through mitochondria and NADPH oxidase under high glucose conditions^23,24^. These findings demonstrate two-way negative feedback between ROS and TXNIP, and reduced TRX acts as an intermediate^20^. In response to intracellular ROS accumulation, TXNIP shuttles into mitochondria via unclear mechanisms and binds to the disulfide bond of TRX2, inhibiting the reducing functions of TRX2 and oxidizing TRX2 to form the TXNIP/TRX2 complex. TRX2 is dissociated from apoptotic signal-regulated kinase 1 (ASK1) by TXNIP, and the TXNIP/TRX2 complex triggers the generation of mitochondrial ROS and induces ASK1 phosphorylation. Phosphorylated ASK1 induces the cleaved form of caspase 3 by stimulating cytochrome *c* (Cyto *c*) secretion. Thus, oxidative stress-induced TXNIP shuttling promotes the mitochondrial apoptosis pathway, leading to various injuries, including those of mitochondria, cardiac tissues, kidney, and lung^4,9,10^.

### TXNIP induces the ASK1-mediated apoptotic signaling pathway

**Fig. 1:**
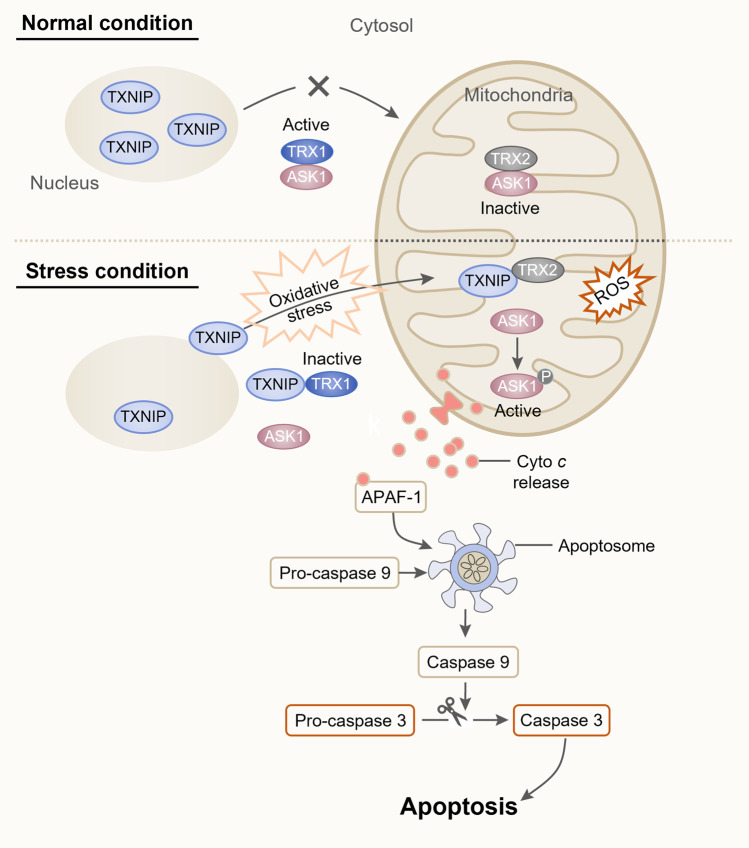
Schematic representation of oxidative stress-induced subcellular TXNIP shuttling and the apoptosis pathway. Under normal conditions, TXNIP is primarily localized in the nucleus. This allows TRX2 in the mitochondria to remain bound to ASK1, inhibiting the activation of ASK1 and promoting cellular survival. In response to oxidative stress, TXNIP shuttles to the mitochondria, where it competes with ASK1 for TRX2 binding, generating a TXNIP/TRX2 complex and leading to ASK1 phosphorylation. p-ASK1 stimulates Cyto *c* release into the cytosol, which activates Apaf-1. Apaf-1 oligomerizes into an apoptosome complex. Pro-caspase 9 is recruited to the apoptosome and is activated by autocleavage. Activated caspase 9 cleaves pro-caspase 3 and leads to a programmed apoptosis pathway.

TXNIP may induce glucocorticoid-related apoptotic signals by antagonizing the antiapoptotic effect of TRX via intracellular ROS scavenging^3^. TRX promotes antiapoptotic functions by binding to and inhibiting the proapoptotic protein ASK1, which is a member of the mitogen-activated protein (MAP) kinase family^25^. In response to oxidative stress, TXNIP translocates to the cytosol and/or mitochondria, while it is localized in the nucleus under basal conditions. Cytosolic TXNIP binds to TRX1 and dissociates ASK1 from TXR1, leading to activation of the p38 mitogen-activated protein kinase (p38 MAPK) signaling pathway^26^. Moreover, in mitochondria, TXNIP binds to mitochondrial TXR2 and prevents the reducing actions of TRX2, resulting in the activation and phosphorylation of ASK1^10^. ASK1 is generally bound to TRX2 under normal conditions; however, under oxidative stress and subsequent translocation of TXNIP to the mitochondria, ASK1 dissociates from TRX2, inducing an apoptotic signal cascade. Disruption of the ASK1-TRX2 interaction induces the phosphorylation of ASK1 and the release of Cyto *c* from the mitochondria into the cytosol. Cyto *c* activates Apaf-1 by directly binding to it, leading to its oligomerization into the apoptosome complex, which recruits pro-caspase 9 and initiates autocatalytic cleavage prior to pro-caspase 9 separation from the apoptosome and becoming inactive^27^. Cleaved caspase 9 can directly activate caspase 3 by cleaving pro-caspase 3, which leads to a programmed apoptosis pathway^4,28^ (Fig. 1).

TXNIP regulates the survival of pancreatic β-cells, which are considerably sensitive to oxidative stress due to their low expression levels of antioxidant enzymes such as TRX and manganese superoxide dismutase (MnSOD), and β-cell apoptosis plays a key role in type 1/2 diabetes^29^. TXNIP overexpression in β-cells activates apoptosis primarily by stimulating the mitochondrial death signaling pathway involving caspase 3 cleavage, Cyto *c* release, and phosphorylation/activation of ASK1^10,29^. On the other hand, TXNIP depletion has protective effects and enhances β-cell survival even under high glucose conditions by improving AKT/Bcl-xL signaling^30^. Recently, Chen et al. identified a novel regulatory mechanism of the CCAAT/enhancer binding protein homologous protein (CHOP)-dependent TXNIP signaling pathway in nephrotic syndrome (NS). Albuminuria-induced ER stress promotes the CHOP-TXNIP signaling axis, leading to activation of the mitochondrial p-ASK1-mediated intrinsic apoptosis pathway^9^. Several in vitro models have demonstrated the contribution of TXNIP to apoptosis, including retinal pericytes exposed to high glucose and endothelial cells stressed by palmitate^31,32^. Thus, TXNIP-related apoptosis is considered a potential target in models of various disorders^13^.

### TXNIP activates the NLRP3 inflammasome by mediating ER stress

**Fig. 2:**
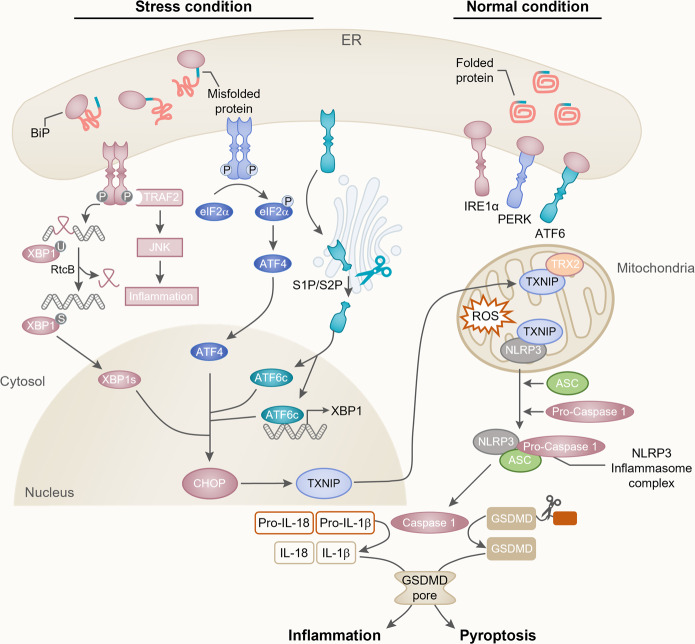
ER stress-induced CHOP/TXNIP/NLRP3 inflammatory pathway. The UPR is induced by the accumulation of misfolded proteins in the ER. Disassembly of BiP from IRE1α, PERK, and ATF6 during ER stress activates each domain. Activated IRE1α induces the nonconventional splicing of X-box binding protein-1 (xbp1u; unspliced form) mRNA and forms xbp1s mRNA, which encodes XBP1s (a transcriptional activator). PERK is phosphorylated by ER stress, causing the phosphorylation of eIF2α. This process prevents ribosome assembly, which leads to translational inhibition and allows the cell to deal with ER stress. However, ATF4 dissociates translation inhibition during stress conditions and induces CHOP activation. Under stress conditions, ATF6 is translocated to the Golgi compartment and cleaved by S1P/S2P enzymes. Cleaved ATF6 (ATF6c) induces CHOP and promotes the expression of XBP1s. CHOP induces TXNIP shuttling from the nucleus to the mitochondria, which is required for the generation of mitochondrial ROS. The induction of CHOP/TXNIP signaling activates the NLRP3 inflammasome. Ultimately, active caspase 1 stimulates the maturation of IL-1β/IL-18 and GSDMD cleavage, leading to inflammation and pyroptosis-associated pathways.

TXNIP plays an essential role in cellular metabolism by activating inflammatory signaling via NLRP3^6^. The initiation of inflammatory signaling requires the formation of inflammatory sensors (known as inflammasomes). Inflammasomes are multiprotein complexes consisting of NLRP3 oligomers, ASC, and pro-caspase 1^30^. NLRP3 contains three domains: the pyrin domain (PYD), leucine-rich repeat domain (LRR), and nucleotide binding/oligomerization domain (NACHT)^33^. ASC contains two domains: the caspase recruitment domain (CARD) and PYD. Pro-caspase 1 is composed of the CARD and the p10/30 domain^33^. NLRP3 inflammasome activation is initiated by the stimulation of nuclear factor-κB (NF-κB), which translocates to the nucleus under oxidative stress conditions. NF-κB, which controls the transcriptional induction of NLRP3 and proinflammatory cytokines, including pro-IL-1β and pro-IL-18, was shown to provide the first step in the activation of the NLRP3 inflammasome^33^. The second step of NLRP3 inflammasome activation is initiated by the direct interaction between TXNIP and NLRP3 in a redox-related manner. Proinflammatory pathway activation and ROS accumulation induce the interaction of TXNIP with NLRP3 and ASC via the pyrin domain of NLRP3, and subsequently, the CAR domain of ASC recruits and interacts with pro-caspase 1^32^. In addition, these stress conditions promote the separation of TXNIP from TRX2, which enables TXNIP to bind directly with NLRP3 in the mitochondria^9,34^. These interactions lead to the formation of the NLRP3 inflammasome and induce the cleavage of pro-caspase 1, which results in the production of activated caspase 1. Caspase 1 processes pro-IL-18 and pro-IL-1β into their mature forms (IL-18 and IL-1β) and mediates their secretion into the cytosol, which facilitates the inflammatory reaction. Active caspase 1 cleaves gasdermin D (GSDMD) within the linker between the N- and C-terminal domains^35^. The N-terminal fragment shuttles to the plasma membrane and oligomerizes to form membrane pores with diameters of 10–20 nm^35^. The membrane pores promote the release of the inflammatory cytokines IL-1β/IL-18, membrane rupture, and cell swelling, eventually leading to pyroptosis^7^ (Fig. 2).

Several studies have reported that the induction of TXNIP by ER stress promotes the activation of the NLRP3 inflammasome^7^. The unfolded protein response (UPR) is initiated by the stimulation of three ER transmembrane proteins located in the ER: activating transcription factor 6 (ATF6), protein kinase RNA (PKR)-like ER kinase (PERK), and inositol-requiring enzyme-1α (IRE1α)^36^ (Fig. 2).

ATF6 is a type 2 transmembrane protein that contains cytosolic and luminal domains. The activation of ATF6 by ER stress is dependent on binding immunoglobulin protein (BiP) (also known as glucose-regulated protein (GRP78)) binding^36^. Under normal conditions, BiP is located in the luminal domain of ATF6^37^. UPR activation releases ATF6 and results in its Golgi localization. ATF6 disulfide bonds are remodeled under ER stress, which induces ATF6 translocation to the Golgi apparatus, where it is cleaved by site-1 and site-2 proteases (S1P and S2P)^37^. ATF6c, the cytosolic domain of ATF6, translocates into the nucleus and stimulates the transcription of UPR-related genes by directly binding to the ER stress response component or by interacting with nuclear transcription factor Y (NF-Y)^38^. ATF6 mostly encodes ER stress-related transcription factors such as X-box binding protein 1 (XBP1), ER chaperones, and ER-associated protein degradation (ERAD) components^36^. It is thought that because ATF6 directly encodes X-box binding protein-1, the activation of XBP1 can enhance CHOP expression^39^. In addition, ER stress induced by albuminuria can stimulate ATF6 and lead to increased CHOP levels^9^. Elevated expression of CHOP results in the overexpression of TXNIP, which ultimately activates the NLRP3 inflammasome, similar to PERK and IRE1α signaling.

IRE1α is a type 1 transmembrane protein with a kinase and endoribonuclease domain. After being activated, IRE1α phosphorylates and dimerizes, activating its RNase domain, which catalyzes the deletion of a 26-nucleotide intron with the mRNA sequence of XBP1^40^. This splicing step is accomplished by the RtcB protein, which ligates spliced XBP1 mRNA, allowing the translation of XBP1s^40^. XBP1s is a major regulator of genes involved in ERAD and protein folding. Additionally, the kinase domain of IRE1α can couple ER stress with inflammation. The kinase IRE1α activates TNF receptor-associated factor 2 (TRAF2) and c-Jun N-terminal kinase (JNK) signaling modules, which can initiate inflammatory responses^41^. IRE1α can thus regulate TXNIP/NLRP3 inflammasome activity. ER stress-induced IRE1α overexpression activates CHOP by stimulating XBP1s or JNK phosphorylation by ASK1^9,41^. In addition, an increase in IRE1α can activate TXNIP by reducing the activity of microRNA-17 (miR-17), which is a TXNIP-destabilizing miRNA.

PERK, a type I transmembrane kinase, is activated by ER stress. PERK activation induces phosphorylation of the eukaryotic initiation factor 2α (eIF2α) subunit, which inhibits protein synthesis^42^. This process decreases the excess proteins entering the ER in stressed cells and permits the selective translation of the mRNA encoding transcription factor 4 (ATF4)^38^. Sustained ATF4 expression by eIF2α phosphorylation under ER stress allows the induction of CHOP and CHOP-dependent TXNIP/NLRP3 inflammasome activation^42,43^. Thus, the novel regulatory mechanism of TXNIP/NLRP3 inflammatory signaling via PERK activation represents a convergence point of different stress pathways governed by a specific regulator.

Overall, the stimulation of these three signal transducers by ER stress typically regulates the activity of CHOP and TXNIP. The induction of CHOP/TXNIP signaling activates the NLRP3 inflammasome and ultimately caspase 1 to stimulate the maturation and release of IL-1β and IL-18^41^. Thus, CHOP/TXNIP signaling is a core regulator of NLRP3 inflammasome activation and the apoptosis/pyroptosis pathway and may act as a key mediator linking an enormous variety of deleterious stimuli such as oxidative stress and inflammation.

## Role of TXNIP in the pathogenesis of diseases

### Diabetes

Diabetes is a common metabolic disorder characterized by hyperglycemia, loss of functional pancreatic β-cell mass, and insufficient production of the glucose-lowering hormone insulin^44,45^. Moreover, diabetes can cause secondary complications, including kidney failure, nerve damage, blindness, and cardiovascular disease^46^. Pancreatic β-cells play an important role in maintaining glucose levels by sensing blood glucose levels and secreting insulin^26^.

TXNIP is the most upregulated gene in human pancreatic islet cells exposed to high levels of glucose^47,48^. Glucose leads to dose- and time-dependent recruitment of carbohydrate response element-binding protein (ChREBP) to the TXNIP promoter, which regulates TXNIP transcription in vivo in human islets, as well as INS-1 β-cells^49^. In contrast, forkhead transcription factor O1 (FOXO1) competes with ChREBP to bind to the TXNIP promoter in vivo in human islets and INS-1 cells. FOXO1 significantly decreases glucose-induced TXNIP expression by blocking glucose-induced ChREBP binding to the TXNIP promoter^48^. Mammalian target of rapamycin (mTOR) also decreases β-cell TXNIP expression by inhibiting ChREBP transcriptional activity. β-cell-specific mTOR deficiency increases the expression levels of TXNIP and ChREBP, leading to severe reductions in β-cell survival, β-cell mass, islet size, and insulin secretion in streptozotocin (STZ)-induced diabetic mice^50^.

The induction of TXNIP in the islets of human diabetic patients and diabetic mice causes β-cell apoptosis^47,50,51^. TXNIP overexpression by high glucose exposure was accompanied by caspase 3 pathway-mediated β-cell apoptosis in INS-1 cells and the isolated islets of mice and humans. Glucose toxicity-induced β-cell apoptosis was attenuated in TXNIP-deficient mouse islets^51^. Furthermore, TXNIP deficiency prevented mitochondrial β-cell death by activating antiapoptotic AKT/Bcl-xL signaling and protected against type 1 and type 2 diabetes by maintaining β-cell mass and function^30^. Therefore, TXNIP plays a pivotal role in β-cell survival, function, and glucose homeostasis.

The proinflammatory cytokine IL-1β is associated with β-cell dysfunction and death, leading to insulin deficiency in both type 1 and type 2 diabetes^8,47^. Glucose-induced TXNIP is essential for activation of the NLRP3 inflammasome pathway. TXNIP binds to NLRP3 and activates the NLRP3 inflammasome, resulting in caspase 1 activation and IL-1β production^8,34^. Furthermore, TXNIP is a critical link between ER stress and inflammation in β-cell death. High glucose-induced ER stress increases TXNIP expression, which promotes ER stress-mediated NLRP3 inflammasome activation, IL-1β production, and β-cell death^8^. TXNIP deficiency impairs NLRP3 inflammasome activation and IL-1β secretion. *Txnip*^-/-^ and *Nlrp3*^-/-^ mice have similar phenotypes with improvements in glucose tolerance and insulin sensitivity compared to wild-type mice after eight weeks of a high-fat diet feeding^34^. Therefore, the inhibition of TXNIP is a reasonable therapeutic strategy against diabetes.

### Chronic kidney disease

Chronic kidney disease (CKD) is characterized by irreversible functional or structural kidney damage and has emerged as one of the most prominent causes of death worldwide^52,53^. Patients with progressive CKD can have common symptoms such as proteinuria/albuminuria, glomerulosclerosis, and interstitial fibrosis^6,9,53,54^. Interestingly, heavy proteinuria is not only a symptom but also a cause of glomerulosclerosis, interstitial fibrosis, and loss of kidney function^9^. TXNIP expression is significantly increased in human proteinuric kidney disease, including focal segmental glomerulosclerosis (FSGS), membranous nephropathy (MN), and diabetic nephropathy (DN)^9^. Furthermore, upregulation of the ER stress-induced transcription factor CHOP due to albuminuria drives TXNIP shuttling from the nucleus to the mitochondria, which is required for mitochondrial ROS production in *Lamb2*^-/-^ mice, which is an NS model. Mitochondrial-specific TRX2 oxidation by mitochondrial ROS dissociates TXNIP, activates the NLRP3 inflammasome complex and releases ASK 1 to induce mitochondria-dependent apoptosis. Thus, TXNIP inhibition by CHOP deletion suppresses NLRP3 inflammasome activation and p-ASK1-dependent mitochondrial apoptosis, decreasing albuminuria and improving kidney function in NS^9^.

Renal interstitial fibrosis is the final common pathway of progressive CKD^53–55^. TXNIP deletion attenuates renal fibrosis and damage by regulating SMAD3 and p38/ERK MAPK phosphorylation in a unilateral ureteral obstruction (UUO) mouse model of renal interstitial fibrosis. UUO-induced renal inflammation is suppressed by inhibiting the activation of NFκB and NLRP3 inflammasomes and the induction of proinflammatory cytokines IL-1β, IL-18, and MCP1 in the kidneys of *TXNIP*^-/-^ mice^56^. In addition, kidney aging is accompanied by renal fibrosis, which drives CKD^53,55,57^. TXNIP overexpression in tubular epithelial cells increases the expression levels of the cellular senescence markers p16^INK4a^ and γH2AX and the profibrotic factors α-SMA, TGF-β1/SMAD3, and collagen I, which ultimately lead to aging-related renal fibrosis and a decline in kidney function. Furthermore, a study showed that TXNIP directly interacts with signal transducer and activator of transcription 3 (STAT3) and promotes the STAT3 signaling pathway-activated profibrotic response, which was markedly reduced by the STAT3 inhibitor HY-N0174, as well as genetic TXNIP deletion^57^.

DN is a major clinical complication of diabetes and the most common cause of CKD^58,59^. STZ-induced diabetic mice exhibit significant increases in functional and structural kidney damage, including albuminuria, glomerular fibrosis, podocyte foot process effacement, glomerular basement membrane thickening, Nox4-mediated oxidative stress, and IL-1β-mediated inflammation, which are attenuated by TXNIP deletion^58^. Furthermore, high glucose-induced TXNIP upregulation is a key mediator of intracellular ROS generation and apoptosis in cultured human podocytes^58^ and HK-2 human proximal tubular cells^60^. The overexpression of FOXO1, a positive regulator of antioxidant genes, prevents high glucose-induced TXNIP upregulation, TRX downregulation, and ROS accumulation, resulting in reduced apoptosis in proximal tubular cells^60^. Moreover, TXNIP silencing mitigates Bax/Caspase 3 pathway-induced apoptosis by suppressing mTOR and p38 MAPK signaling activation in high glucose-induced cultured podocytes and STZ-induced diabetic kidneys. High glucose-induced podocyte apoptosis is also alleviated by treatment with Raptor and Rictor shRNAs, the mTOR-specific inhibitor KU-0063794, and the p38 MAPK inhibitor SB203580^59^.

Overall, the upregulation of TXNIP was observed and implicated in pathological pathways in vivo and in vitro in the NS model, UUO-induced renal fibrosis model, aging-related renal fibrosis model, DN model, and human proteinuric kidney diseases. Thus, emerging evidence suggests that TXNIP is a key pathological regulator of CKD.

### Neurodegenerative diseases

TXNIP has been implicated in neurodegenerative diseases, including Alzheimer’s disease (AD) and Parkinson’s disease (PD)^6,26^. AD is the most common age-related neurodegenerative disease among the elderly population. In the AD brain, clinical symptoms are accompanied by pathological features, including extracellular amyloid β (Aβ) plaque deposition and abnormal intracellular accumulation of hyperphosphorylated tau^26,61,62^. Continuous accumulation of Aβ and hyperphosphorylated tau in the brain triggers ER stress, oxidative stress, and inflammation, ultimately contributing to neurodegeneration^61^. Several studies have reported a significant increase in TXNIP in the cortex and hippocampus of postmortem human AD brains and an in vivo AD mouse model^61–64^. The upregulation of TXNIP in the AD brain is mostly related to inflammation^6,26^. ER stress induced by phospho-tau and Aβ promotes the TXNIP-induced NLRP3 inflammasome pathway, which leads to IL-1β-induced neuroinflammation in the human AD brain^61,62^. Recently, TXNIP is closely associated with Aβ and receptor for advanced glycation end products (RAGE), which are counterreceptors of proinflammatory ligands such as Aβ in the hippocampus of 5xFAD AD mice. The RAGE-TXNIP axis induces Aβ translocation to mitochondria, leading to mitochondrial dysfunction and oxidative stress, which induce NLRP3 inflammasome activation, IL-1β secretion, and the cleavage of the pyroptotic protein GSDMD. Thus, TXNIP silencing blocks NLRP3 inflammasome-induced IL-1β secretion and GSDMD activation in the hippocampus of 5xFAD AD mice^63^.

PD is the second most common neurodegenerative disease and is characterized by α-synuclein accumulation and dopaminergic neuron loss^65,66^. TXNIP was significantly increased in human α-synuclein-overexpressing A53T mice and α-synuclein-transfected HEK293 cells. TXNIP overexpression impairs lysosomes by downregulating the critical lysosomal membrane protein ATP13A2, resulting in α-synuclein accumulation. Increased TXNIP in the mouse substantia nigra causes dopaminergic neuron loss^65^. Treatment with MPP^+^ , a dopaminergic neurotoxin, leads to TXNIP-mediated NLRP inflammasome activation and pyroptosis^66^. Collectively, these studies demonstrate that TXNIP plays a role as a pathological contributor and accelerator in the progression of AD and PD.

## Therapeutic potential of targeting TXNIP in diseases

TXNIP is a key pathological regulator of several diseases, including CKD, diabetes, and neurodegenerative diseases, and is an attractive therapeutic target for the development of novel drugs. Several studies have reported that some small-molecule compounds and phytochemicals can inhibit TXNIP expression and TXNIP-induced signaling pathways. Here, we review a variety of TXNIP inhibitors and discuss the mechanisms by which they inhibit TXNIP directly or indirectly (Table 1).

**Table 1:** Potential inhibitors of TXNIP and their targets.

Name	Target	Disease	Mechanism
Verapamil	Calcium channel, TXNIP	Diabetes	Reduces TXNIP expression by inhibiting ChREBP binding to the TXNIP promoter in HG-induced INS-1 β-cells^45^
	TXNIP, ROS, p38 MAPK	Alzheimer’s disease	Blocks TXNIP expression, ROS production, and p38 MAPK activation in 5xFAD AD mice^68^
	TXNIP, NLRP3	Alzheimer’s disease	Inhibits TXNIP expression and NLRP3 inflammasome activation in 5xFAD AD mice^63^
Metformin	ROS, TXNIP, NLRP3	Diabetes	Prevents ROS production and TXNIP-NLRP3 inflammasome activation by suppressing ER stress in HG-treated adipose tissues^69^
	TRX, TXNIP, NLRP3	Diabetic atherosclerosis	Inhibits the dysregulation of TRX/TXNIP and activation of the NLRP3 inflammasome via AMPK activation in HG-treated THP-1 macrophages^70^
	TXNIP, NLRP3	Intestinal I/R injury	Suppresses TXNIP expression, TXNIP-NLRP3 interaction, and NLRP3 inflammasome activation in mice with intestinal I/R injury^71^
SRI-37330	TXNIP	Diabetes	Inhibits TXNIP promoter activity and TXNIP expression in INS-1 β-cells, mouse islets, and human islets in response to HG^44^
DI-NBP	Nrf2, TRX, TXNIP	Alzheimer’s disease	Enhances TRX activity and blocks TXNIP expression through Nrf2 upregulation, resulting in the inhibition of TXNIP-NLRP3 binding and NLRP3 inflammasome activation in APP/PS1 AD mice^64^
W2476	TXNIP	Diabetes	Blocks TXNIP expression in STZ-induced diabetic mice and HFD-induced obese mice^72^
Diltiazem	Calcium channel, TXNIP	Diabetes	Decreases TXNIP mRNA expression in HG-induced INS-1 β-cells^45^
DMF	ROS, TXNIP, NLRP3	Diabetic vascular disease	Inhibits TXNIP/NLRP3 expression and ROS levels and enhances SOD/Nrf2 activation in STZ-induced diabetic rats^76^
Quercetin & Allopurinol	ROS, TXNIP, NLRP3	Diabetes	Suppresses ROS levels and TXNIP-NLRP3 inflammasome activation in STZ-induced diabetic rats^73^
Salidroside	ROS, TXNIP, NLRP3	Diabetic nephropathy	Inhibits HG-induced ROS levels and TXNIP-NLRP3 inflammasome activation in HBZY-1 glomerular mesangial cells^74^
	ROS, TXNIP, NLRP3	Nonalcoholic fatty liver disease	Blocks ROS production and TXNIP-NLRP3 inflammasome activation through AMPK pathway activation in HFD mice^75^
Resveratrol	ROS, TXNIP, NLRP3	Diabetes	Prevents ROS production and TXNIP-NLRP3 inflammasome activation by suppressing ER stress in HG-treated adipose tissues^69^
Fisetin	ROS, TXNIP, NLRP3	Acute otitis media	Upregulates SOD/Nrf2 expression and Inhibits activation of the MAPKs/NF-κB pathways and the TXNIP-NLRP3 inflammasome in LPS-treated mice^77^

*HG* high glucose, *I/R* ischemia‒reperfusion, *HFD* high-fat diet.

Verapamil, an anti-hypertensive calcium channel blocker, inhibits TXNIP transcription by decreasing the binding of ChREBP to the TXNIP promoter^45,46,67^. Oral administration of verapamil prevents β-cell apoptosis and improves endogenous β-cell function, glucose homeostasis, and insulin sensitivity in diabetic mouse models^45^ and in adults with recent-onset type 1 diabetes^46^. Additionally, verapamil treatment reduces tau phosphorylation at Ser202/Thr205 by blocking the TXNIP/p38 MAPK pathway and preventing Aβ-induced NLRP3 inflammasome activation and IL-1β secretion in the hippocampus of 5xFAD AD model mice^63,68^.

Metformin is widely used as a first-line treatment for type 2 diabetes^67,69–71^. Treatment with metformin prevents TXNIP/NLRP3 inflammasome activation by suppressing ER stress in the adipose tissues of STZ-injected diabetic mice, thereby blocking diabetes-induced adipose dysfunction^69^. Metformin inhibits high glucose-induced TXNIP/NLRP3 inflammasome activation through AMPK activation in macrophages, which suppresses diabetes-mediated atherosclerosis^70^. In addition, metformin suppresses TXNIP expression and the TXNIP-NLRP3 interaction, which protects against intestinal ischemia‒reperfusion injury and pyroptosis via the TXNIP-NLRP3-GSDMD pathway^71^.

SRI-37330 was recently identified as an orally bioavailable, nontoxic, small molecule that suppresses the mRNA and protein expression of TXNIP by inhibiting TXNIP promoter activity in INS-1 cells and mouse and human islets. Oral treatment with SRI-37330 decreased glucagon secretion and activity, reduced hepatic glucose production, and reversed hepatic steatosis in STZ- and obesity-induced diabetic mice^44^.

The antioxidant Dl‐3‐n‐butylphthalide (Dl‐NBP) has recently been used clinically for the treatment of cerebral ischemia. Dl‐NBP treatment enhances TRX activity and reduces TXNIP expression, the TXNIP-NLRP3 interaction, and NLRP3 inflammasome activation by upregulating Nrf2, a major transcription factor that responds to oxidative stress. The Dl‐NBP-mediated signaling pathway mitigates neuronal apoptosis and Aβ plaque-associated microglial dystrophy in the brains of APP/PS1 AD model mice^64^.

Oral administration of the small-molecule W2476, which is a modulator of TXNIP expression, rescues STZ-induced diabetic mice by promoting β-cell survival and insulin secretion^72^. Similarly, inhibiting TXNIP expression with the natural flavonoid quercetin and antioxidant allopurinol improves liver inflammation and lipid accumulation in STZ-induced diabetic rats^73^.

The phytochemical salidroside, which is isolated from the herbal plant *Rhodiola rosea*, alleviates high glucose-induced cell proliferation, oxidative stress, and ECM accumulation in HBZY-1 rat glomerular mesangial cells by inhibiting TXNIP-NLRP3 inflammasome pathway activation^74^. Salidroside administration also attenuates hepatic steatosis and hepatic inflammation in high-fat diet-fed mice by inhibiting the AMPK-dependent TXNIP/NLRP3 pathway^75^.

In addition, several other small molecule compounds, such as diltiazem^45^ and dimethyl fumarate (DMF)^76^, as well as other phytochemicals, such as resveratrol^69^ and fisetin^77^, have shown inhibitory effects on TXNIP expression and TXNIP-induced signaling pathways.

## Conclusion and future perspective

We reviewed currently available information on the roles of the multifunctional protein TXNIP in the pathogenesis of diseases such as diabetes, chronic kidney disease, and neurodegenerative disease, and summarized a variety of TXNIP inhibitors. Emerging evidence suggests that TXNIP is a promising therapeutic target, and its inhibition protects against the development of various diseases. According to previous studies, CHOP inhibition, FOXO1 overexpression, and ChREBP inhibition for targeting the TXNIP promoter to inhibit TXNIP expression could be used to develop effective TXNIP inhibitors and novel drug candidates. Moreover, targeting TXNIP could have a secondary effect through pharmacological inhibition of the NLRP3 inflammasome for inflammatory disease treatment because TXNIP is essential for activation of the NLRP3 inflammasome pathway. This review may be helpful to better understand the pathogenic roles of TXNIP in various diseases to develop effective therapeutic strategies.
